# Performance, safety and efficiency comparison between 10,000 and 5000 cuts per minute vitrectomy using a 25G cutter: a prospective randomized controlled study

**DOI:** 10.1186/s40942-023-00452-1

**Published:** 2023-03-21

**Authors:** Nicholas S. K. Fung, Anthony K. H. Mak, Marten Brelen, Chi Wai Tsang, Shaheeda Mohamed, Wai Ching Lam

**Affiliations:** 1 Department of Ophthalmology, Grantham Hospital, Hong Kong, China; 2 Department of Ophthalmology, Li Ka Shing Medical School, The University of Hong Kong, Hong Kong, China; 3 Department of Ophthalmology and Visual Sciences, The Chinese University of Hong Kong, Hong Kong, China; 4 Department of Ophthalmology, University of British Columbia, 2550 Willow Street, Room 301, Vancouver, BC V5Z 3N9 Canada

## Abstract

**Purpose:**

This study aims to compare the performance of the 25+® UltraVit® 5000 cuts per minute (cpm) vitrectomy probe versus the 25+ ® Ultravit 10,000 cpm® beveled tip, dual drive vitrectomy probe.

**Method:**

In this prospective randomised controlled clinical trial, 52 eyes of 52 consecutive patients were randomized into either the 10,000 cpm (25 patients) or 5000 cpm vitrectomy group (27 patients). Patients were evaluated preoperatively, intraoperatively, and postoperatively on the first day, and at 1 week, 1 month and 3 months. The main outcome measures were vitrectomy time, and secondary endpoints were time to induction of posterior vitreous detachment, intraoperative complications, and number of instruments used.

**Results:**

The vitrectomy time was shorter in the 10,000 cpm group (413.7 s) compared to the 5000 cpm group (463.4 s), although there was no significant difference (p = 0.5999).

One patient had an iatrogenic retinal break in the 10,000 cpm group while two patients had an iatrogenic retinal break in the 5000 cpm group. The time for posterior vitreous detachment (PVD) induction and the number of instruments used were not significantly different between the two groups.

**Conclusion:**

The difference in vitrectomy times between the 10,000 cpm vitrectomy probe and the 5000 cpm cutter were not statistically significant. This may suggest that other factors affect efficiency rather than the limitations of equipment.

## Highlights

- Vitrectomy time may be limited by surgeon factors rather than instrument factors when using Ultra high-speed vitrectors
- There are no significant differences in surgical time between 5000 and 10,000 cpm vitrectors
- Faster cutters may result in fewer introperative complications.

## Introduction

Three-port pars plana vitrectomy instrumentation has undergone rapid advances since its first introduction in the early 1970s. Whilst various aspects such as viewing system, endo-illumination and vitrectomy machines have all been extensively refined over the years, a significant proportion of the developments have focused on the vitrectomy cutter [1]. The flow rate and tractional forces generated by the cutter in vitrectomy surgery are influenced by parameters such as cutter speed, aspiration rate, gauge size, port size, and duty cycle [2]. Numerous developments have focused on these factors, in order to improve efficiency and enhance the safety of vitrectomy surgery. Recently, the Advanced Ultravit® High-Speed (UHS) bevelled probe was introduced, which can deliver 10,000 cuts per minute (cpm) with a dual pneumatic drive technology. This high-speed probe is available in 23, 25, and 27 gauge, and its novel bevelled tip design has reduced the distance between the cutting port and retina to a mere 0.23 mm (0.009 inches) for all 3 gauges. Compared to standard flat-tipped probes, the reduction of retina-to-port distance is between 40 and 55% depending on the gauge size. This allows the surgeon to achieve closer access to tissue planes, which can be particularly useful, for example, when cutting vitreous close to the retina in cases which need vitreous base shaving. In the past, vitreous cutters employing spring return mechanism suffered from lower duty cycle (percentage of time the probe port remains open) as cut rate increased [3]. On the other hand, the dual pneumatic drive technology has separate air lines for opening and closing of the port. This allows duty cycle to be modulated independently of cut speed [4, 5], and achieve a high cut rate without compromising on efficiency as the duty cycle can be maintained. Moreover, the larger port opening size with the bevelled cutter design complements the high cut rate by maintaining the efficiency of vitreous cutting and removal at a high cut rate.

The main benefits of high cutter speed are two-fold: shorter vitrectomy time and safer surgery. At a higher cut rate, vitreous is segmented into smaller pieces, leading to reduced viscosity within aspiration tubing and lower resistance of vitreous flow [6]. This can improve efficiency of vitreous removal and shorter vitrectomy time [6–8]. Faster cut rate can also reduce turbulence and traction on the adjacent retina within the sphere of influence of the vitrectomy cutter. This is due to a reduction in the amount of uncut vitreous entering the port and improved fluidic stability [9]. Rizzo et al. compared 25 gauge vitrectomy between 1500 cpm versus 5000 cpm, and reported that iatrogenic retinal breaks occurred at a rate of 21.7% and 1.7% respectively [7]. Multiple clinical studies have compared the safety and efficacy between different gauge sizes of vitrectomy cutters. Studies have also compared different cutter speed and investigated their fluidics behaviour under experimental conditions [3, 5, 10]. Few studies, however, have systemically evaluated the safety and efficiency profile of different cutter speeds in clinical settings [7, 8]. To our knowledge, there has yet to be a study comparing 25 gauge 5000 cpm and 10,000 cpm vitrectomy.

## Method

This was a prospective, interventional, multi-surgeon, randomized controlled trial aiming to compare the current 25+ gauge 5000 cpm UHS vitrector with the 25+ gauge 10,000 cpm vitrector from Alcon Constellation® Vision System (Alcon Laboratories, Inc, Fort Worth, TX).

Consecutive patients over the age of 18 requiring vitrectomy for vitreous haemorrhage (VH), epiretinal membrane (ERM), macular hole (MH), dislocated lens, rhegmatogenous retinal detachment (RRD) and diabetic tractional retinal detachment were randomized into the study from January 2019 till August 2019 after written consent was obtained from all participants. Randomization was achieved by simple randomization using a computer generated random table while recruitment and assignments were done by a research assistant blind to the procedures and follow up. Patients with ocular comorbidities affecting surgical view including corneal opacities or scar, previous vitrectomy, history of trauma or requiring silicone oil were excluded from the study. Written informed consents were obtained from all participants, and the study was approved by the hospital and university institutional review board before the study commenced (IRB ref: UW 18-179). The study was registered with clinialtrials.gov (NCT04859556) and The University of Hong Kong Clinical Trials Centre (HKU-CTC1786). All methods adhered to the tenets of the Declaration of Helsinki (version 2000) guidelines for research involving human subjects and ICH-GCP on protocol.

### Study procedures

All surgeries were performed by experienced vitreoretinal surgeons (WCL, NSKF). The primary endpoint was recorded as core vitrectomy time determined from the duration the vitrector was activated by the Alcon Constellation® Vision System. Posterior vitreous detachment (PVD) induction was manually timed from the moment of aspiration until detachment was achieved with or without the assistance of triamcinolone. Secondary endpoints included the number of surgical instruments used, and time taken to induce a PVD. The Constellation® vitrector system offers 3 settings of duty cycle: “core” mode with maximum port opening to achieve high flow rates; “shave” mode with minimum port opening to allow flow rates; and “50/50” mode with 50% port opening time and 50% port closure time. In the current study, vitrectomy was done for all cases with “core” mode duty cycle control and a proportional vacuum setting (variable aspiration from 0 to 650 mmHg) at the highest fixed cut rates of either 5000 cpm or 10,000 cpm for the UltraVit 25G+ vitrectomy probes. Instruments used during surgery included 25G light pipe, vitrectomy cutter, forceps, scissors, endolaser, endo-diathermy and soft tip backflush needle.

Medical history, ophthalmic examination, intraocular pressure and best corrected visual acuity (BCVA) were recorded at the initial visit before surgery, then at 1 month and 3 months post-surgery. BCVA was measured using Early Treatment Diabetic Retinopathy Study charts and converted to logarithm of minimum angle of resolution (logMAR).

### Sample size justification

C Mariotti et al. 2016 reported the mean ± standard deviation of core vitrectomy time was 161.32 ± 39.10 s in the 25 gauge (G) 7500 cpm Group and 184.10 ± 41.69 s in the 25G 5000 cpm Standard Group [8]. The observed difference in mean core vitrectomy duration between subjects treated with 7500 cpm probes and those in the Standard Group was 22 s. With the assumption that the higher cutting speed (10,000 cpm) will take at least 33 s less time in doing the core vitrectomy than 5000 cpm group, at one sided 0.05 significance level, with a common standard deviation 40.4 s, 24 patients at each group will have 80% power to achieve the study primary objective.

All statistical analysis was performed using the Prism 8 by Graphpad (ver 8.2.1). Averages were compared using the unpaired T Test with a statistical significance level of P < 0.05.

## Results

Fifty-two eyes in 52 patients were included (25 in the 10,000 cpm group and 27 in the 5000 cpm group). There were 21 females and 31 males. Diagnoses included were 14 vitreous hemorrhage, 17 epiretinal membrane, 15 retinal detachment (including x tractional retinal detachment), 3 macular hole, 3 dislocated lens. There were no significant differences between the two groups at baseline. (Table 1).

**Table 1:** Baseline characteristics

	10K group (N = 25)	5K group (N = 27)	p-value
Age	62.48	64.96	0.3776
Male	12 (48%)	16 (59%)	0.4257
Diagnosis			0.5002
Vitreous hemorrhage	7 (28%)	7 (26%)	
Macular hole	1 (4%)	2 (7%)	
Epiretinal membrane	7 (28%)	10 (37%)	
Retinal detachment	8 (32%)	7 (26%)	
Dislocated IOL	2 (8%)	1 (4%)	
Right eye	15 (60%)	14 (52%)	0.5635

Unpaired T-test, statistically significant *p < 0.05

**Fig. 1:**
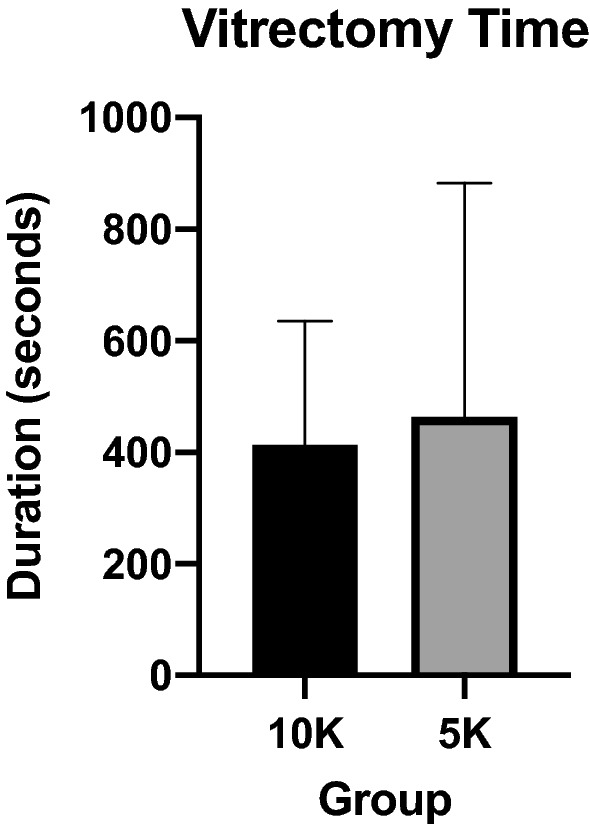
Comparision of vitrectomy time in seconds between 10K and 5K groups

**Fig. 2:**
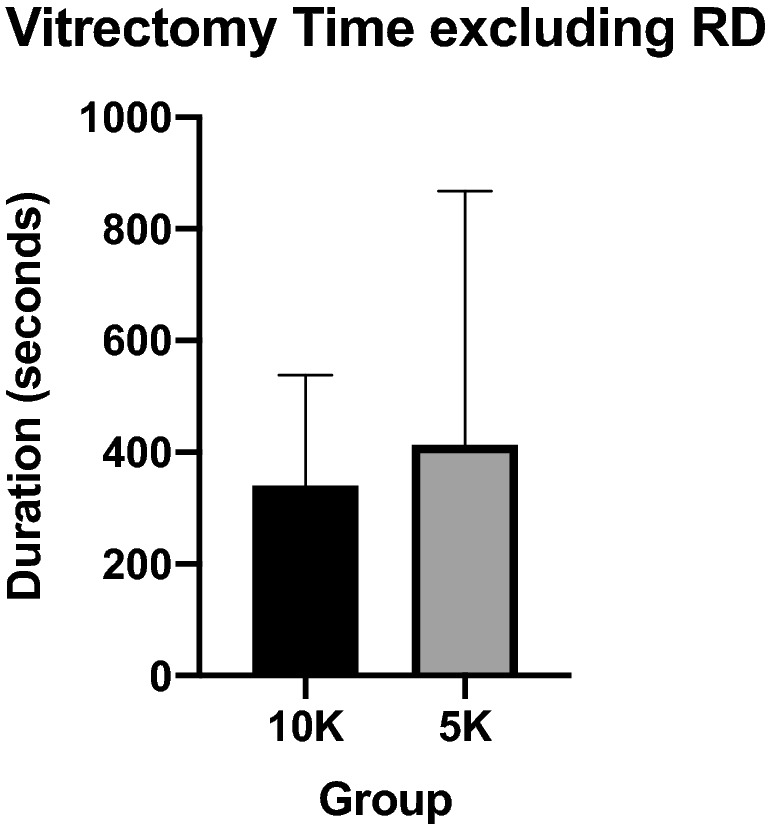
Comparison of vitrectomy time in seconds between 10K and 5K groups excluding retinal detachment cases

Mean vitrectomy time was 413.7 ± 221.4 s in the 10,000 cpm group compared to 463.4 ± 419.4 s in the 5000 cpm group, no significant difference found because of the large standard deviation (p = 0.5999) (Fig. 1). The difference between means was 49.69 ± 94.12 s (95% CI − 139.4 to 238.7). A subgroup analysis (n = 37) excluding retinal detachment patients also showed the same trend of 340.6 ± 197 s in the 10,000 cpm group compared to 413.7 ± 453.9 s in the 5000 cpm group, but did not show any statistical significance (p = 0.5421) (Fig. 2). The difference between means was 73.11 ± 118.8 s (95% CI − 168.0 to 314.2).

The vitrectomy times varied between different surgical procedures (Table 2), with a significant difference seen between retinal detachment (585.9 ± 230.6 s) compared to vitreous haemorrhage (384.5 ± 220.5 s, p = 0.0349) and ERM (274.6 ± 106.0, p = 0.0002) (Table 3).

**Table 2:** Vitrectomy time

	Combined time (s)	10 K group time (s)	5 K group time (s)	P value*
Vitreous hemorrhage	384.5 ± 220.5	457.7 ± 255.5	335.8 ± 223.4	0.9287
Macular hole	338.0 ± 178.0	258.0 ± 0	378.0 ± 231.9	0.9994
Epiretinal membrane	274.6 ± 106.0	240.0 ± 77.73	298.9 ± 119.8	0.9984
Retinal detachment	585.9 ± 230.6	569. 0 ± 196.9	605.3 ± 279.4	0.9999
Dislocated IOL	311.0 ± 100.6	324.0 ± 138.6	285.0 ± 0	> 0.9999

T-test between sub group 10K vs 5K

**Table 3:** Subgroup analysis (p-value)

	Vitreous hemorrhage	Macular hole	Epiretinal membrane	Retinal detachment
Macular hole	0.9948			
Epiretinal membrane	0.4585	0.9839		
Retinal detachment	0.0349*	0.2442	0.0002*	
Dislocated IOL	0.9711	0.9998	0.9980	0.1595

Subgroup multiple comparison by one way ANOVA & Tukey’s test, *Significance p < 0.05

The best corrected visual acuity improved significantly from 1.44 logMAR preoperatively to 0.91 logMAR (p < 0.001) at the first month and to 0.87 logMAR (p < 0.001) at the third month after surgery.

In the 10,000 cpm group, one peripheral iatrogenic retinal break was noted in a tractional retinal detachment patient who required membrane dissection with curved scissors and forceps. In the 5000 cpm group, retinal breaks were found in two cases, a MH and an ERM peel. In the macular hole patient, 3 superior breaks were noted after peripheral shave vitrectomy. In the ERM patient, there was one single break at 2 o’clock position noted on indentation without any subretinal fluid. Both cases were successfully treated with endolaser alone. Importantly, in both groups, there were no iatrogenic retinal breaks during vitrectomy in rhegmatogenous retinal detachment with a mobile retina. None of the cases in either group needed gas tamponade in addition to the barrier laser applied to the iatrogenic breaks.

There were no post-operative complications, such as hypotony, endophthalmitis or retinal detachments, noted in any patients at final follow up. Time to PVD induction was not significantly different between groups due to small numbers, as most patients already had PVD but the mean duration in the 10,000 cpm group was 80 s, and 27 s in the 5000 cpm group. There were no significant differences in the number of instruments used between the two groups, 4.4 in the 10,000 cpm group vs 3.96 in the 5000 cpm group (p = 0.627).

## Discussion

In the past, per Poiseuille’s law (Flow rate = ΔPπr^4^/8η*L* where ΔP is the pressure difference across the length of the probe, r is the inner radius of the probe, η is the viscosity, and *L* is the length of the probe), flow rate was reduced relative to the inner diameter of the lumen of the vitrectomy probe [9]. With the introduction of the dual pneumatic drive technology, first introduced by Alcon’s Constellation® in 2008, extending the duty cycle allowed longer port opening times and faster cut rates. There is a slight difference in terms of duty cycle with the two cutter designs. The 5000 cpm cutter has a duty cycle of 50/50 at maximum speed, which is consistent with our study setting, while the 10,000 cpm cutter has a duty cycle of 56/44 (open/closed) ratio [1]. The flow rate also differs with the 25+ cutters where 5000 cpm cutter is 2.59 cc/min and the 10,000 cpm cutter is 2.76 cc/min. The faster cut rate allowed increased vitreous flow despite smaller gauge probes using the same vacuum aspiration [11]. Our study supports this and demonstrated a trend that higher speed cutters may be more efficient and result in shorter vitrectomy times, although the results did not achieve statistical significance. This may be due to the fact that compared to older 25G Accurus cutter systems (1500 cpm with single-actuation spring-return pneumatic drive with no duty cycle control), the newer 25G+ 5000 cpm cutter systems have already significantly reduced vitrectomy times due to the ability to maintain high flow rates, and a more substantial change in vitrectomy time may be needed with the 25+ 10,000 cpm cutter to show statistical significance. For example, in a study in 2011 by Rizzo et al. the average vitrectomy time was 1583 s for the Alcon Accurus® at 1500 cpm and the dual drive line Alcon Constellation at 5000 cpm was 1106 s, compared to 463 s and 413 s for the Alcon Constellation 5000 cpm and 10,000 cpm 25G cutters respectively in this study [7]. In the study by Marrioti et al., the average vitrectomy time for the 5000 cpm group was much faster at 184 s while using the same equipment. [8] The large range of vitrectomy times in the three studies for 5000 cpm cutter also suggests that vitrectomy time is highly variable and dependent on multiple factors, such as surgeon practice, variable operation parameters of duty cycle, aspiration rate, and characteristics of the aspiration medium. With faster and more efficient equipment, the vitrectomy time is more likely to be limited by the surgeon factor rather than the vitrectomy cutter design. In addition, the difference of 40 s between the two groups may not affect surgery time in practice. The authors find that rather than being limited by the cutter speed, the technique of seeking vitreous and the constant movement of the cutter to different areas of the eye can improve efficiency.

In our study, the vitrectomy times between different types of cases were significantly different, such as between retinal detachment and ERM or VH. Although attempts were made to standardize the vitrectomy technique, individual surgeon preference and technique of the various surgeons may also have affected vitrectomy time as the surgeons are necessarily not masked to the cutter. However, having multiple surgeons also increases generalisability of the study findings in a real-world situation.

Besides achieving shortened vitrectomy times with the higher cut rates, the safety profile is ultimately more important. It is shown that at a faster cut rate, there is decreased flow per opening cycle and therefore less vitreous traction and increased fluidic stability, which may be reflected in the fewer iatrogenic retinal breaks in our study [12]. There was also no retinal detachment postoperatively with a follow up of 3 months in the current study. The bevelled tip design and shorter port-to-retina distance with improved fluidic stability allowed for safer vitreous removal with confidence, especially with detached retina, allowing more complete vitrectomy. The true benefits of the new cutter in terms of safety may therefore only be seen with a longer follow up. Furthermore, the bevelled tip design allows for multi-purpose functions including blunt dissection, pick action, adhesion cutting and even membrane peeling. Although the number of instruments used in this study was not different in the 5000 cpm and 10,000 cpm cutter groups, the probe advancements with the 10,000 cpm beveled tip cutter can reduce need for ancillary instrumentation, allowing faster and safer surgery due to reduced instrument exchanges, and less iatrogenic breaks from vitreous base dragging, but this may not be reflected in the study results of vitrectomy times as the vitrectomy machine is unable to differentiate core vitrectomy from adhesion dissection. Indeed, these advantages may have skewed the calculation of vitrectomy times to be longer in some cases. The difference in designs eventually did not have an impact on the instruments used between cases as expected, the individual preference and need for each case is highly variable. The bevel tipped design did allow for closer to retina work but for complex tractions, the need for additional forceps and scissors were irreplaceable. Not surprisingly, the PVD induction was not significantly different between the two groups as the instrument gauge and aspiration were identical. More cases without existing PVD would be needed to assess whether the bevel tip can help in this area.

Rizzo et al. showed that a simple modification to the inner duct of the cutter would create a second channel for vitreous to be aspirated and cut, effectively doubling the cut rate and optimizing the duty cycle, similar to the finding with the dual port designs from Lima et al. [13, 14] The commercial form is seen with the introduction of the dual blade 2-dimensional cutter launched by DORC TDC Continuum® (Dutch Ophthalmic Research Center, Netherlands) in 2015 where the port effectively remains constantly open and results in the same aspiration at 2000 cpm as at 16,000 cpm with the same sized gauge. Alcon has also announced their new 20,000 cpm HyperVit® Dual Blade with modifiable duty cycle continuously open port, bevelled tip cutter which is available in both 25+G and 27+G. Bausch + Lomb has also introduced their dual blade system for the Stellaris Elite® called the Bi Blade®, available in 23G, 25G and 27G which will cut up to 15,000 cpm. As the vitrector probe cutting rates increase, the benefits might be expected to plateau due to Poiseuille’s law unless technology optimizes flow even further.

Limitations of this paper include the small number of cases, heterogeneity of cases and surgeons, which may lead to greater variation and standard deviation in vitrectomy times, as well as short follow up time. Moreover, although all surgeons were experienced vitreoretinal surgeons, there might also be a possible learning curve as well as surgeons’ preference to the extent of core vitrectomy, differences in the ocular disease and vitreous status, surgical setting, etc. which can impact the vitrectomy times. Still, the new bevelled tip design with the port closer to the tip of the 10,000 cpm vitrector was instantly noticeable with greater confidence in shaving vitreous close to the retina, especially in detached cases due to increased stability of the retina from the decreased pulsatile traction. There is likely an adaptation period to adjust individual habits and preferences in order to use new equipment to its fullest potential. Future studies may benefit from using a more homogenous patient group, blinding of the surgeon when probes use identical designs such as the 20,000 cpm HyperVit dual blade, and further standardising the procedures to specify the extent of vitreous removal.

## Conclusion

In conclusion, the 10,000 cpm 25+ gauge vitrectomy probe showed similar vitrectomy times and safety profile when compared to the 5000 cpm cutter. It is likely that even at higher cutting speeds, the efficiency may be limited to other factors such as surgeon preferences and ocular disease. In addition, the reduced port-to-retina distance, bevelled design, and larger vitrector port opening, along with improved duty cycle control and control over aspiration, have expanded the cutter versatility, and can improve the efficiency and safety of small gauge vitrectomy by reducing traction and turbulence. Future studies with fewer variables and a more homogenous patient group may help to demonstrate the differences between the 10,000 cpm over 5000 cpm vitrectomy cutters.

## Data Availability

Data available by request to corresponding author up to 3 years after publication.
